# Association of Altered Plasma Lipidome with Disease Severity in COVID-19 Patients

**DOI:** 10.3390/biom14030296

**Published:** 2024-03-01

**Authors:** Zhengzheng Zhang, Naama Karu, Alida Kindt, Madhulika Singh, Lieke Lamont, Adriaan J. van Gammeren, Anton A. M. Ermens, Amy C. Harms, Lutzen Portengen, Roel C. H. Vermeulen, Willem A. Dik, Anton W. Langerak, Vincent H. J. van der Velden, Thomas Hankemeier

**Affiliations:** 1 Metabolomics and Analytics Centre, Leiden Academic Centre for Drug Research, Leiden University, 2333 CC Leiden, The Netherlands; z.zhang@lacdr.leidenuniv.nl (Z.Z.); naama@timacs.org (N.K.); a.s.d.kindt@lacdr.leidenuniv.nl (A.K.); m.singh@lacdr.leidenuniv.nl (M.S.); l.lamont@lacdr.leidenuniv.nl (L.L.); a.c.harms@lacdr.leidenuniv.nl (A.C.H.); 2 Tasmanian Independent Metabolomics and Analytical Chemistry Solutions (TIMACS), Hobart, TAS 7008, Australia; 3 Department of Clinical Chemistry and Hematology, Amphia Hospital, 4818 CK Breda, The Netherlands; avangammeren@amphia.nl (A.J.v.G.); aamermens@gmail.com (A.A.M.E.); 4 Department of Population Health Sciences, Institute for Risk Assessment Sciences, University Utrecht, 3584 CK Utrecht, The Netherlands; l.portengen@uu.nl (L.P.); r.c.h.vermeulen@uu.nl (R.C.H.V.); 5 Laboratory Medical Immunology, Department of Immunology, Erasmus MC University Medical Center Rotterdam, 3015 GD Rotterdam, The Netherlands; w.dik@erasmusmc.nl (W.A.D.); a.langerak@erasmusmc.nl (A.W.L.); v.h.j.vandervelden@erasmusmc.nl (V.H.J.v.d.V.)

**Keywords:** SARS-CoV-2, COVID-19, lipidomics, lipids, cytokine, inflammation

## Abstract

The severity of COVID-19 is linked to an imbalanced immune response. The dysregulated metabolism of small molecules and bioactive lipids has also been associated with disease severity. To promote understanding of the disease biochemistry and provide targets for intervention, we applied a range of LC-MS platforms to analyze over 100 plasma samples from patients with varying COVID-19 severity and with detailed clinical information on inflammatory responses (>30 immune markers). This is the third publication in a series, and it reports the results of comprehensive lipidome profiling using targeted LC-MS/MS. We identified 1076 lipid features across 25 subclasses, including glycerophospholipids, sterols, glycerolipids, and sphingolipids, among which 531 lipid features were dramatically changed in the plasma of intensive care unit (ICU) patients compared to patients in the ward. Patients in the ICU showed 1.3–57-fold increases in ceramides, (lyso-)glycerophospholipids, diglycerides, triglycerides, and plasmagen phosphoethanolamines, and 1.3–2-fold lower levels of a cyclic lysophosphatidic acid, sphingosine-1-phosphates, sphingomyelins, arachidonic acid-containing phospholipids, lactosylceramide, and cholesterol esters compared to patients in the ward. Specifically, phosphatidylinositols (PIs) showed strong fatty acid saturation-dependent behavior, with saturated fatty acid (SFA)- and monosaturated fatty acid (MUFA)-derived PI decreasing and polystaturated (PUFA)-derived PI increasing. We also found ~4000 significant Spearman correlations between lipids and multiple clinical markers of immune response with |R| ≥ 0.35 and FDR corrected Q < 0.05. Except for lysophosphatidic acid, lysophospholipids were positively associated with the CD4 fraction of T cells, and the cytokines IL-8 and IL-18. In contrast, sphingosine-1-phosphates were negatively correlated with innate immune markers such as CRP and IL-6. Further indications of metabolic changes in moderate COVID-19 disease were demonstrated in recovering ward patients compared to those at the start of hospitalization, where 99 lipid species were altered (6 increased by 30–62%; 93 decreased by 1.3–2.8-fold). Overall, these findings support and expand on early reports that dysregulated lipid metabolism is involved in COVID-19.

## 1. Introduction

The coronavirus (COVID-19) pandemic has presented a significant global challenge due to the rapidly growing number of new variants, and this highlights the urgent need for the characterization of SARS-CoV-2 pathogenicity and host immune response [1]. Patients with COVID-19 experience a spectrum of clinical symptoms of differing severity, ranging from asymptomatic to critical pneumonia, acute respiratory distress syndrome (ARDS), and death [2]. Furthermore, patients exhibit high inter-individual variability in response to SARS-CoV-2 infection, which makes it difficult to identify those at risk of adverse outcomes. The involvement of lipids in COVID-19 is far reaching. Coronaviruses first react with the host cell membrane for entry and infection, and this has brought novel insights into the involvement of cellular lipids [3,4,5,6,7]. Lipids are the main building blocks of cell membranes and play a crucial role in the viral replication process, affecting the host lipid metabolism [4,8]. Membrane lipids also release precursors of eicosanoids and docosanoids, polyunsaturated fatty acids (PUFAs), to regulate the immune response during an infection [9]. Several lipidomic studies have described an altered lipidome profile in COVID-19 patients [10,11,12,13]. In an early pandemic study, patients with varying severity of COVID-19 (compared to healthy subjects) showed decreased serum levels of sphingolipids, glycerophospholipids, and choline, while phosphocholine was increased [10]. Another study reported decreased plasma diacylglycerols (DGs) and increased levels of sphingomyelins (SMs) and monosialodihexosyl gangliosides (GM3s) in COVID-19 patients compared to healthy controls [11]. Suggesting an overall alteration in the lipid balance in COVID-19 patients, additional studies have reported decreased serum total cholesterol, HDL, and LDL alongside increased triglycerides (TGs) [12,13]. Another study showed that comprehensive lipid mapping unveils host dependency factors that remain consistent among various SARS-CoV-2 variants [14].

Previously, we reported the discovery of COVID-19 plasma perturbations in amines which reflected inflammation and oxidative stress [15], and we also suggested that altered signaling lipid metabolism reflects excessive immune response and disrupts the resolution of inflammation [16]. To further study lipidome changes in COVID-19 patients, we analyzed 103 plasma samples from 44 patients with varying disease severity and conducted a comprehensive profiling of over 1000 lipids by targeted LC-MS/MS. The measured lipids underwent differential analysis based on disease severity (i.e., hospitalization status) and were also correlated with over 30 immune response markers obtained for the same cohort [17]. The results of this study contribute to the gathering evidence of lipid alteration in COVID-19 patients and provide further insight into the cellular mechanisms involved in the progression of the disease.

## 2. Materials and Methods

### 2.1. Cohort

In total, 44 patients and 103 blood samples were included in a previous cohort study [14,15], and the key clinical parameters are summarized in Table 1 and Table S1. SARS-CoV-2 infection status was confirmed by PCR.

### 2.2. Sample Collection

The study collected EDTA blood samples at regular intervals of 3–4 days throughout the study period (Table S1). A small portion of the collected blood was immediately used for flow cytometry immune profiling, while the remaining samples were kept on ice and processed to plasma within 2 h. The plasma samples were then divided into smaller portions and stored at −20 °C until serological analysis or transportation to the analytical chemistry laboratory. At the laboratory, the samples were stored at −80 °C until sub-aliquoting and analysis.

### 2.3. Sample Analysis

The flow cytometric leukocyte analysis and serological analysis of cytokines and soluble cell surface molecules were performed as in previous studies [14,15,17]. We applied comprehensive lipidomics profiling platforms covering glycerolipids, glycerolphospholipids, sphingolipids, and sterols [18,19]. The data quality was checked by an in-house quality control software (mzQuality) (version 1.0.0) using study QC replicates, blank samples, and internal standards. A total of 1076 lipid features with RSDQC < 30% measured by the two platforms passed quality control and were utilized in the statistical analysis. The individual measurement techniques used in this study are comprehensively described in Supplementary Document S1.

### 2.4. Statistical Analysis 

In total, 37 cytokine and immune markers as well as 1076 lipid features were used and cuberoot-transformed before statistical analysis. No imputations were performed on the lipid features due to the zero missingness. A linear regression correcting for age, sex, and BMI was applied to distinguish between the changes in ICU and ward patients. Paired analyses between start and end stages from the same patient were then performed using a paired *t*-test assuming unequal variances. The lipid fold change (FC) was calculated based on the untransformed data. The correlations between lipids and immune markers were performed on both ICU and ward patients. The Benjamini–Hochberg method was applied for the FDR correction for the *p*-values obtained in all tests with a significance cutoff of |R| ≥ 0.35 and Q < 0.05. All statistical analyses and visualization were performed using R (version 4.0.3) packages (ggpubr and stats).

## 3. Results

### 3.1. Altered Lipid Profiles in COVID-19 Patients in the Ward and ICU

During the first wave of COVID-19, and before the improvements made to the wards of hospitals during the later waves, the more severely affected patients were transferred to the ICU for more intensive care, mechanical ventilation, and generally improved monitoring. The location of the patient is thus a proxy for severity of COVID-19. To determine the potential relationships between plasma lipids and disease severity, we profiled 25 plasma samples from 7 patients in the ICU and 78 plasma samples from 37 patients in the ward using targeted lipidomics approaches. A description of the COVID-19 patient cohort, with samples collected at varying hospitalization days, is summarized in Table 1 and further detailed in Table S1. 

**Figure 1:**
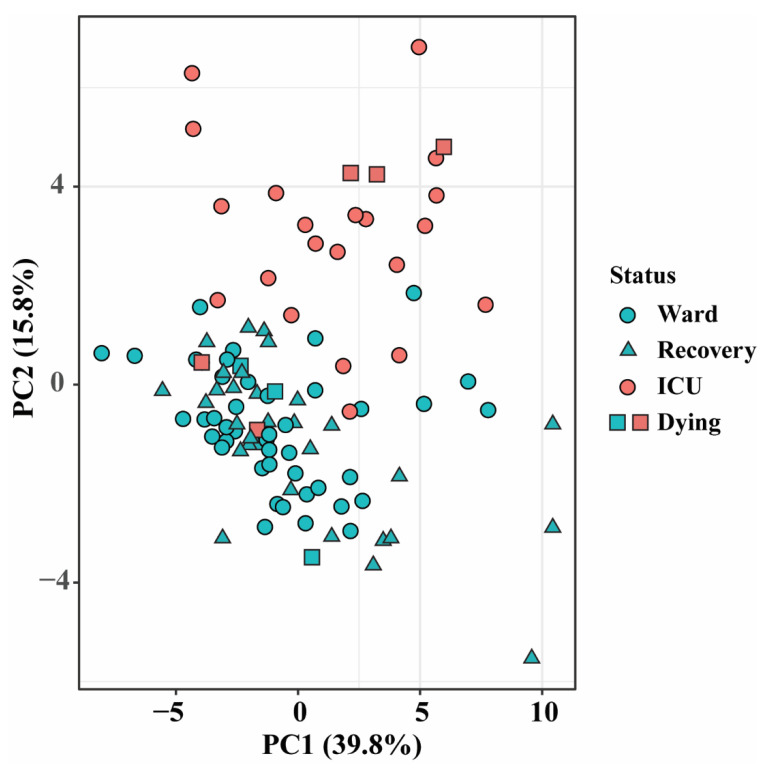
PCA scores plot of samples from patients admitted to the ward (blue markers) or ICU (red markers), based on all lipid features data (cuberoot-transformed and Pareto-scaled). Data points of samples taken within a day of release from hospital (“recovery”) are depicted by triangles, and samples taken within 4 days of death are indicated by squares.

**Figure 2:**
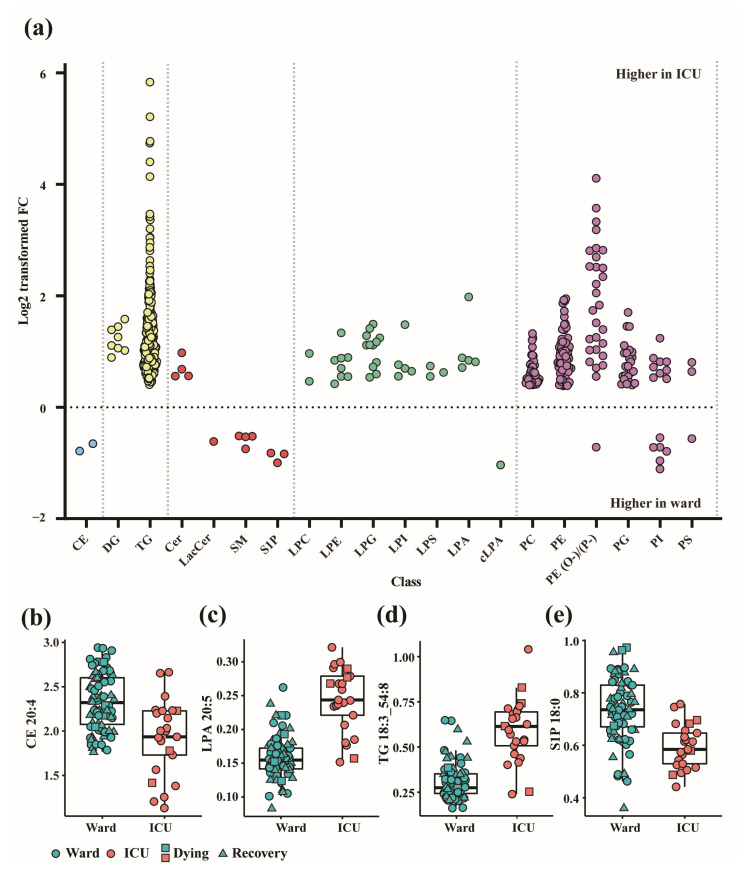
(**a**) Distribution of log2-transformed fold changes of 531 significantly changed lipid features with FC ≥ 1.3 or ≤0.7) and FDR Q value < 0.05 in ICU and ward patients. Different colors represent different lipid (sub)categories: light blue: CE; yellow: glycerolipids; red: (glyco)sphingolipids; green: (lyso)phospholipids; purple: phospholipids; (**b**–**e**) Box and whisker and scatter plots of lipids (Q < 0.005) differentiating between hospitalization status: ICU (red) vs. ward (blue). Data points of samples taken within a day of release from hospital (“recovery”) are depicted by triangles, and samples taken within 4 days of death are indicated by squares. Prior to plotting, lipid peak area ratios with internal standards were cuberoot-transformed. Lipids: (**b**) CE 20:4; (**c**) LPA 20:5; (**d**) TG 18:3_54:8; (**e**) S1P 18:0. The detailed results are in Table S3.

In order to visualize the distribution of this cohort, all 1076 lipid features that passed the quality control process were first analyzed using a principal component analysis (PCA), which showed some separation between the ward and ICU patients (Figure 1). Next, a linear regression model for univariate analysis was employed, adjusting for age, sex, BMI, and count of samples per patient, to identify the most important biomarker candidates distinguishing the patients in the ward from those in the ICU. A total of 531 lipids (including the ratio of sphinganine-1-phosphate (Spha1P) 18:0/sphingosine-1-phosphate (S1P) 18:1) across 19 lipid classes with fold changes ≥ 1.3 or ≤0.7 and FDR Q values < 0.05 were considered significant (Table S3), indicating a widely changed lipidome in severe COVID-19 patients. All significantly changed species in the lipid classes TG, ceramides (Cer), DG, lysophoshatidylcholines (LPC), lysophosphatidylethanolamines (LPE), lysophosphatidylglycerols (LPG), lysophosphatidylserines (LPS), lysophosphatidylinositols (LPI), phosphatidylcholines (PC), alkyl phosphatidylcholines PC(O-), phosphatidylglycerols (PG), phosphatidylethanolamines (PE), and lysophosphatidic acids (LPA) increased, while all significantly changed species in the lipid classes cholesteryl esters (CE), lactosylceramides (LacCer), S1P, and SM decreased in the ICU patients when compared to the ward patients, as is shown in the FC plot (Figure 2a) and in the specific examples (Figure 2b–e). All PS and alkyl phosphatidylethanolamines (PE (O-)) classes increased in the ICU patients, except for two AA- precursor-containing species, PS 18:1/20:4 and PE O-16:0/20:4, which were decreased in the ICU patients. The PIs showed strong fatty acid saturation-dependent behavior, with SFA- and MUFA-derived PI decreasing and PUFA-derived PI increasing in the ICU patients. Among all these differences, TGs and PE (O-)/alkenyl phosphatidylethanolamines (PE (P-)) showed the biggest increased levels, with FCs up to 57-fold in the ICU patients (Figure 2a; Table S3).

### 3.2. Paired Analysis in Ward Patients

**Figure 3:**
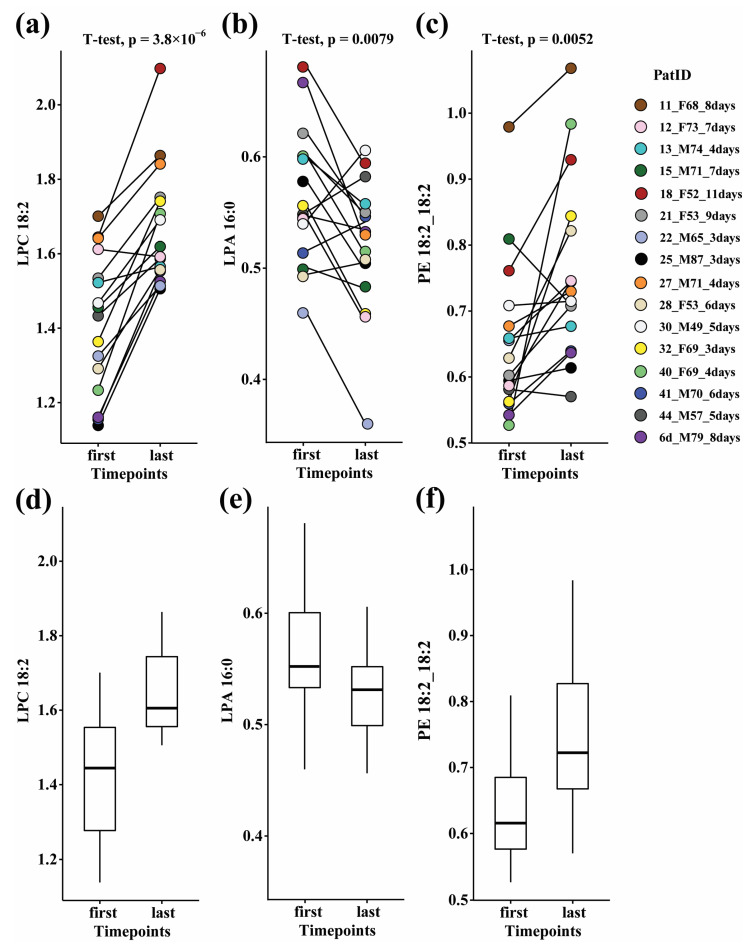
Scatter plots (**a**–**c**) and box and whisker plots (**d**–**f**) of paired changes in lipid levels in COVID-19 ward patients. A line connects each patient’s paired samples, with the first time point being not more than 4 days from admission, and last time point occurring during the 24 h before release from hospital. Lipids: (**a**,**d**) LPC 18:2; (**b**,**e**) LPA 16:0; (**c**,**f**) PE 18:2_18:2. The legend shows each individual patient by marker color and indicates the patient’s number, sex, age, and the number of days between time points. Patient information is provided in Table S2. FDR-corrected paired *t*-tests, gender differences, and fold changes are provided in Table S5.

To quantify the metabolic changes in recovering COVID-19 patients, a paired analysis was performed on samples taken from 16 patients in the ward, hence reflecting moderate disease only. The patients were selected based on sample availability at the start of hospitalization (days 1–4 since admission) and towards the end of hospitalization (within 1 day of release from the hospital), with a minimum interval of three days between the two time points. The analysis was restricted to ward patients, since there were not enough relevant samples available from the ICU patients. In contrast with the widely changed lipidome profiles in patients in the ICU vs. those in the ward, here only 99 lipids were significantly altered (FC ≥ 1.3 or ≤0.7 and FDR Q value < 0.05) as patients neared recovery in the ward (Table S4). Most of the significant changes were found in phospholipids and lysophospholipids, which increased as patients neared recovery (see examples in Figure 3). It is worth noting that other lipid classes, especially TG and SM, remained mostly unchanged throughout hospitalization in the ward, suggesting their higher relevance to severe disease.

### 3.3. Correlation between Lipids and Immune Response Markers

**Figure 4:**
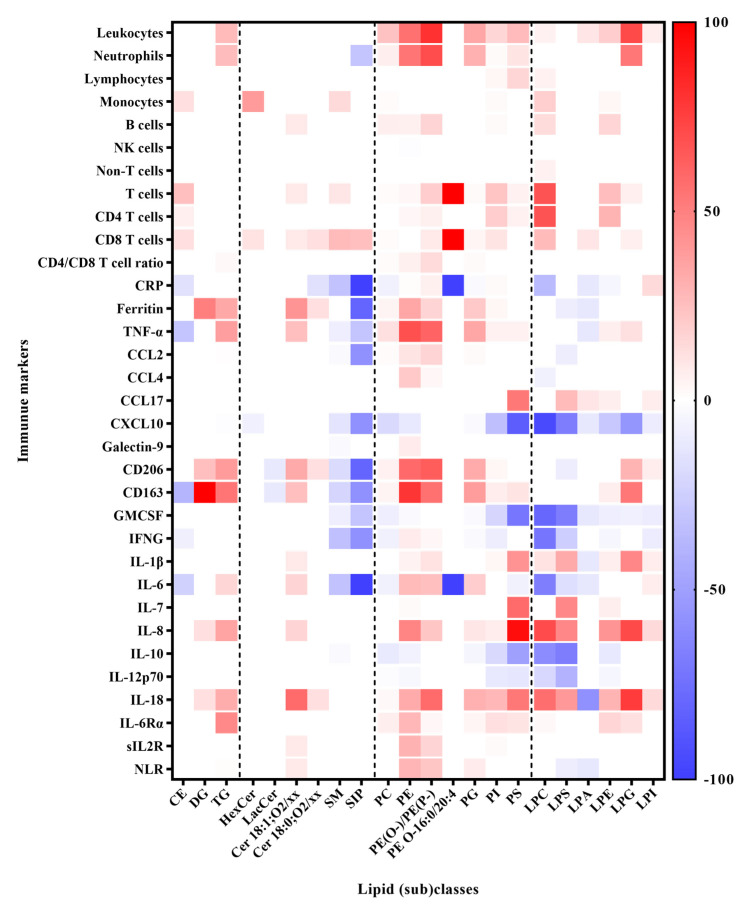
Heatmap of Spearman correlation results for metabolites and immune response markers (all cuberoot-transformed). The color bars represent the percentages of the lipid species with significant correlations, i.e., |R| ≥ 0.35 and Q < 0.05 per class, red for positive correlations and blue for negative correlations. Complete correlation matrices (with R, *p*, and Q values) are provided in Table S5.

**Figure 5:**
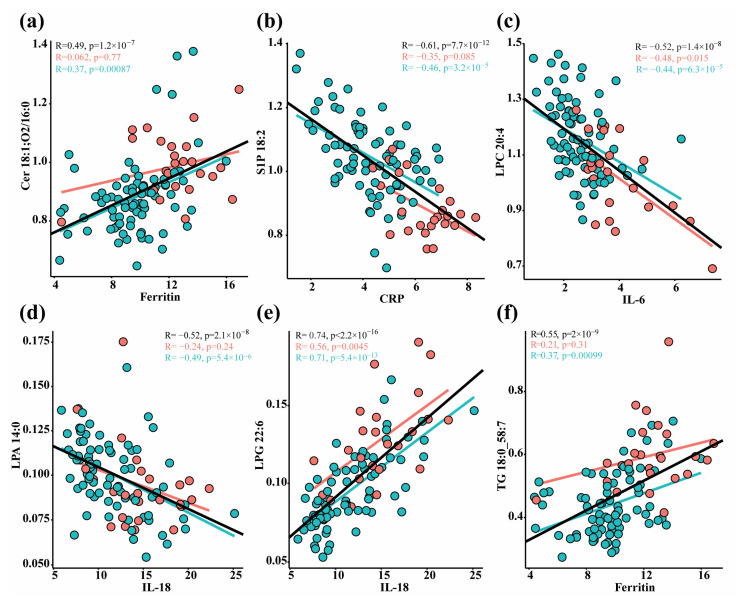
Selected Spearman correlation scatter plots for metabolites and immune response markers (cuberoot-transformed). Red markers are samples from ICU patients and blue markers are from ward patients. The regression lines and Spearman R values and p values (uncorrected) are in black for all samples; red represents ICU patients and blue represents ward patients. Lipids: (**a**) Cer 18:1;O2/16:0 vs. Ferritin; (**b**) S1P 18:2 vs. CRP; (**c**) LPC 20:4 vs. IL6; (**d**) LPA 14:0 vs. IL18; (**e**) LPG 22:6 vs. IL18; (**f**) TG 18:0_58:7 vs. Ferritin; The full correlation results are in Table S5.

The concentration of 37 immune response markers, including different leukocytes, chemokines, cytokines, and others, were also measured (Table S2). We performed a Spearman correlation analysis on the lipid and cytokine data from the whole dataset, and a heatmap summarizing the correlation results of each immune marker with each lipid class is presented in Figure 4. In total, 3995 significant correlations were observed with a threshold of |R| ≥ 0.35 and Q < 0.05, among which were 37 with strong associations, i.e., |R| ≥ 0.6 and Q < 0.05 (Table S5). We observed that SM and S1P were negatively correlated with clinical indices of systemic inflammation including pro-inflammatory markers IL-6, CRP, TNFα, neutrophils, CCL2, GM-CSF, CXCL10, IFNG, and macrophage-activation markers (soluble (s) CD206 and CD163), and positively correlated with the CD8 fraction of T cells (Figure 4; Table S5). Ceramides were positively correlated with IL-6, IL-8, IL-18, CD206, CD163, CD8, TNFα, ferritin, and T cell count. Lysophospholipids except for LPA were positively correlated with IL-18, IL-8, and IL-7 and negatively correlated with CXCL10 (IP10). Selected plots with strong correlations (|R| ≥ 0.60 and Q < 0.05) are shown in Figure 5. The correlations of S1P with immune markers were found to be mostly driven by the dramatic and consistent differences in lipid levels between patients in the ICU and those in the ward (Figure 5b), while the LPLs and TGs were independent of the hospitalization status (Figure 5c–f). The results of the correlation analysis are further utilized in the biochemical Section 4.

## 4. Discussion

Our study reveals noteworthy changes in the lipidome profiles associated with COVID-19 disease severity, including sphingolipids, glycerolphospholipids, and glycerolipids. The biochemical processes relevant to the changes in measured lipids and various immune response markers and their correlations are discussed here.

### 4.1. Sphingolipids Metabolism

Sphingomyelin itself does not have a direct link to COVID-19. However, sphingolipids are essential components of membrane lipid rafts, which mediate signal transduction and immune activation processes [20,21]. Specifically, two protein targets of COVID-19, ACE2 and TMPRSS2, are embedded in lipid rafts and can actively participate in viral infection [22,23]. On the other hand, lipoproteins, including those carrying sphingomyelin, play a role in the body’s immune response. COVID-19 affects multiple systems, including the cardiovascular and immune systems, and lipoproteins are involved in both. Recent studies have shown that lipoproteins, especially high-density lipoproteins (HDLs), may play a protective role against severe complications from COVID-19. HDL particles are known to possess anti-inflammatory and antioxidant properties that can help modulate the immune response and potentially mitigate the severity of inflammatory reactions, such as those seen in severe COVID-19 cases. Sphingomyelin, as a component of these lipoproteins, indirectly contributes to their functions. However, the exact relationship between the sphingomyelin within lipoproteins and COVID-19 is not yet fully understood and remains an area of ongoing research. In our study, we observed increases in ceramide levels and decreases in SM and LacCer in severe COVID cases (ICU) which were consistent with previously reported serum lipid alterations in COVID-19 patients [19]. Ceramides exhibited positive correlations with markers of macrophage activation (CD163 and CD206) that typically increase during the innate immune response in COVID-19 patients. IL-18 was also correlated with ceramides, and this aligns with macrophage activation, as IL-18 can be produced by cells like macrophages. IL-18 plays a role in maintaining the Th1 inflammatory response to viral infection, and it induces the downstream production of IFNG [24]. It has been suggested that IL-18 is associated with a lower risk of developing severe COVID-19 [25]. Ceramides also positively correlated with IL-8, which mediates the inflammatory reaction in the respiratory system (as demonstrated in COVID-19 [26]), promoting neutrophil activation. Positive associations of ceramides with ferritin levels suggest an interplay between iron metabolism and reactive oxygen species (ROS) production during the COVID-19 disease process [27,28]. Ferritin is a surrogate marker for a hyper-immune response, and an elevation in ferritin occurs when intracellular iron concentration and the production of hepcidin, which can be an indicator of cellular damage, increase [28]. As expected, SM levels that were higher in patients in the ward compared to those in the ICU showed negative correlations with an array of immune response markers that characterize hyper-inflammation (CRP, macrophage activation markers, CXCL10, TNF-α, IL-6). This further strengthens the link between SMs and better health outcomes. 

The gathered results may also be linked to the active role of sphingolipids in the development of enveloped viruses at the early stage [3,29,30], which leads to cell apoptosis and immunoescape by lipid raft remodeling [31,32,33,34]. The ceramide–sphingomyelin signaling system plays a central role in the viral infection of human epithelial cells [35]. Sphingomyelins are derived from ceramides in cell membranes via the activity of sphingomyelin synthase. During an adaptive immune response, the membrane-embedded inert sphingomyelins will be hydrolyzed by acid sphingomyelinase (aSMase) and cause the rapid and transient formation of ceramides, which is a hallmark of adaptive responses and cellular repair [30]. This has been shown to be important in COVID-19 as antidepressants have been used to diminish the production of ceramide levels via inhibiting the aSMase levels and preventing SARS-CoV-2 binding in cell models [30]. Furthermore, ceramides have been described as important biomarkers for metabolic disease, e.g., cardiovascular disease [36] and diabetes [37], which are co-morbidities associated with a worse outcomes in COVID-19 patients.

### 4.2. Sphingoid Base 1-Phosphates

Sphingoid base 1-phosphates (S1Ps) are important immune modulators [38] and can be found both in plasma and blood cells. They are part of the sphingolipid signaling cascade, and they play a role in immune cell trafficking and endothelial function, depending also on the expression of specific cell surface receptors [39]. S1Ps have been described as prognostic markers for COVID-19 outcome; a lower circulating level of S1Ps in severe patients suggest the loss of the protective effects of S1Ps [40,41]. S1P 18:1 is the most researched metabolite in this group [42,43], and its extracellular S1P gradient regulates the excretion of lymphocytes such as mature dendritic cells from/to lymphoid organs (low S1P concentration) and into the blood (high S1P concentration) [38,44,45]. This occurs via the binding of S1P to receptors such as S1PR1 on the surface of T cells, while the receptor expression is induced by endothelial chemokines (CXCL10, etc.) [46]. S1Ps have been reported to be related to inflammation resolution, for example via secretion from alveolar macrophages in acute lung injury [47]. Our results support the link to an adaptive immune response,; we observed consistent and strong declines in four sphingoid base 1-phosphates in the ICU patients, and a moderate increase in ward patients nearing recovery. Similar observations were reported for S1P in patients with COVID-19 [11,40,48]. Aligning with the proposed beneficial effects of S1P, we found negative correlations between the four metabolites and the acute immune response markers CRP (Figure 5b), TNF-α, IL-6, ferritin, and others, mostly in a homogenous manner across the four metabolites (Table S5). Analogues of sphingosine-1-phosphate have been suggested for low-risk supportive treatment of COVID-19 patients to reduce the inflammatory response, mitigate lung damage, and even lower the viral load [8,31,49,50].

### 4.3. Glycerophospholipids

We observed an extensive increase in glycerophospholipid levels in the ICU patients. Glycerophospholipids (PC, PE, PI, PG, and PS) are major components of cell membranes and play multiple roles in the response to viral infections. Apart from interacting with glycoproteins on the plasma membrane, viruses can also utilize host-derived lipid membranes in their intercellular transmission to conceal and evade the host’s immune system [51,52]. Glycerophospholipids can undergo degradation, producing lysophospholipids and a free fatty acid. Lysophospholipids are immune modulators and are involved in several pathophysiological processes such as cell proliferation, migration, and tumorigenesis [53]. Our study revealed a strong negative correlation between LPCs and leading markers of hyper-inflammation (GM-CSF, CXCL10, IL-6, CRP). This affinity between better health status and higher levels of LPCs is also demonstrated by the increased levels we observed in patients nearing recovery in the ward. Lysophosphocholines (LPCs) produced by phospholipase A2 (PLA2) can be further metabolized by lysophospholipase D/autotaxin (ATX), leading to their conversion to LPA, which is involved in the innate immune response [53]. Indeed, we observed a decrease in LPA in patients nearing recovery in the ward. When LPCs release a PUFA from their sn2 position, it can serve as a precursor for oxylipins, which play a significant role in the regulation of the immune response during viral infection [16]. PUFAs are essential precursors for a diverse array of oxylipins that are stored in an esterified form and later released by enzymes like COX-1, 12-lipoxygenase (12-LOX), and CYPs found in platelets [54]. A significant decrease in the levels of AA-containing precursors was observed in the phospholipids in the ICU patients. Our previous analysis of signaling lipids in the same cohort found that the ICU patients had significantly lower levels of AA compared to the ward patients [16], and this may be linked to metabolic requirements or an altered level of PLA2 activity [48,55]. One study in COVID-19 patients showed significantly decreased plasma phospholipids alongside increased lysophospholipids, which may indicate enhanced activity of PLA2 [2]. In contrast, we observed extensive increases in glycerophospholipids, including PC, PE, PI, PG, and PS, together with the corresponding lysophospholipids. Disagreement between studies can reflect various cohort differences and treatments (80% received chloroquine that can increase phospholipid levels [56]; however, less than 5% received corticosteroids that inhibit PLA2). Other factors include the analytical methods and the choice of blood product for lipidomics analysis. Plasma is preferred over serum, as it prevents a skewed profiling of oxylipins, sphingoid-based compounds, and lysophospholipids, among other lipids altered by coagulation [57].

### 4.4. Glycerolipids and Other Neutral Lipids

Glycerolipids, including TG and DG, showed higher abundance in the plasma of the ICU patients compared to those in the ward. TGs also correlated with markers of innate immune response (TNF-α, neutrophils), markers of macrophage activation, and ferritin, supporting the link to a worse disease state. Typically, triglycerides serve as energy reservoirs for free fatty acids, and the liver is heavily involved in triglyceride metabolism to ensure a steady supply of energy and a proper distribution of lipids throughout the body [58,59]. Individuals with pre-existing conditions such as diabetes and heart disease, which are characterized by elevated triglyceride levels and chronic inflammation, are predisposed to an increased risk of developing severe COVID-19 [60].

Studies have shown hypertriglyceridemia in COVID-19 patients, which highlights the biochemical significance of TGs, potentially indicating elevated adipose tissue lipolysis [61] and liver function abnormalities, as indicated elsewhere [62,63]. In addition, this was also supported by the lower total CE recorded in the ICU patients in our study, which is often observed in patients with liver damage [64]. Another study also found increased TG and decreased CE in patients with severe symptoms or elderly patients, and this is consistent with the hepatic impairment associated with COVID-19 [7]. Beyond a hypermetabolic state or under-nutrition, such differences in plasma lipids between ward and ICU patients can be attributed to various metabolic pathways associated with viral infection and the host immune response [65]. Triglyceride-rich lipoproteins have been associated with innate immunity [66], and all lipoprotein classes can sequester and prevent excessive inflammation [65]. 

Altogether, the evidence presented in this study suggests that viral infection and subsequent hospital treatment have a profound impact on the systemic lipid metabolism in COVID-19 patients. The pathophysiological effects of the disease seem long lasting. Therefore, it is important to monitor the health state of each patient after discharge. The blood lipid profile can provide a sensitive array of markers linked to inflammation and disease severity.

## 5. Conclusions

In conclusion, our study highlights the fact that the plasma lipidome profiles of COVID-19 patients differ at different stages of the disease. Our findings also demonstrate the interplay between pro-inflammatory cytokines and the host metabolism in COVID-19 patients. Despite the relatively small sample size, this work provides insights that could further assist in drug development and the treatment of COVID-19, and which expands the essential resources promoting further research on the viral disease’s pathogenesis. Future investigations should explore the long-term effects of COVID-19 on lipid metabolism, the utility of metabolic changes as prognostic indicators of disease severity or outcome, and the efficacy of metabolic-targeted therapies for treating COVID-19.

## Figures and Tables

**Table 1:** Demographics of the COVID-19 patients in the lipidomics study. Values are *n* (%) or median (full range). Information about comorbidities and medication (4 weeks pre-admission) is missing for 25% of patients (*n* = 12); smoking status is missing for 9 patients; remaining hospitalization days are missing for 2 patients. All individual patient data are available in Table S1.

	Patients (*n* = 44)	Samples (*n* = 103)
Age (years)	73 [49–87]	71 [49–87]
Male (%)	30 (68%)	65 (63%)
BMI	27 [19–42]	27 [19–42]
Diabetes mellitus (DM)	9 (20%)	
Chronic kidney disease (CKD)	3 (7%)	
Cardiovascular disease (CVD)	7 (16%)	
Chronic obstructive pulmonary disease (COPD)	8 (18%)	
Past smoker	17 (39%)	
Pre-admission beta-blockers, ACE inhibitors	14 (32%)	
Pre-admission corticosteroids	8 (18%)	
Pre-admission chloroquine	27 (61%)	
Days with symptoms till hospitalization	8 [1–19]	
Total hospitalization days	7 [2–62]	
Admitted to ward	37 (84%)	78 (76%)
Admitted to ICU	7 (16%)	25 (24%)
Organ failure	7 (16%)	
Deceased	9 (20%)	
Unfavorable outcome (ICU or death)	12 (27%)	36 (35%)
Invasive breathing support (intubated)	6 (14%)	
Post-admission chloroquine	35 (80%)	
Post-admission corticosteroids	2 (5%)	
Post-admission antibiotics	38 (86%)	
CRP, mg/L (normal < 10)		104.5 [3–577]
IL6, pg/mL (normal < 8)		19.3 [1–397]
Ferritin, ng/mL (normal 10–400)		1035 [84–4807]
Leukocytes, 109/L (normal 4.5–11)		8 [4–20.5]
Lymphocytes, 109/L (normal 0.8–5.0)		0.95 [0.26–3.15]
Neutrophils, 109/L (normal 1.7–6.5)		6.36 [2.3–17.5]
Age (years)	73 [49–87]	71 [49–87]
Male (%)	30 (68%)	65 (63%)
BMI	27 [19–42]	27 [19–42]
Diabetes mellitus (DM)	9 (20%)	
Chronic kidney disease (CKD)	3 (7%)	
Cardiovascular disease (CVD)	7 (16%)	
Chronic obstructive pulmonary disease (COPD)	8 (18%)	
Past smoker	17 (39%)	

## Data Availability

All data utilized in the statistical analyses are available in the Supplementary Files.

## Supplementary Materials

The following supporting information can be downloaded at: https://www.mdpi.com/article/10.3390/biom14030296/s1, Document S1: Method details; Table S1: Description of the cohort patients; Table S2: Immune marker measurement results; Table S3: Significance values for metabolites differentiating between ICU and ward; Table S4: Paired *t*-tests comparing a first and last time point of selected ward patients; Table S5: Spearman correlation coefficients and FDR Q values between metabolites and immune markers.
